# Secretome and immune cell attraction analysis of head and neck cancers

**DOI:** 10.1007/s00262-024-03809-z

**Published:** 2024-09-09

**Authors:** Tara Muijlwijk, Niels E. Wondergem, Fatima Ekhlas, Naomi Remkes, Dennis N. L. M. Nijenhuis, Lennart Fritz, Sonja H. Ganzevles, Iris H. C. Miedema, C. René Leemans, Jos B. Poell, Ruud H. Brakenhoff, Rieneke van de Ven

**Affiliations:** 1 Otolaryngology/Head and Neck Surgery, Amsterdam UMC, Vrije Universiteit Amsterdam, De Boelelaan, 1117-Zh 2A60, 1081 HV Amsterdam, Netherlands; 2 Cancer Center Amsterdam, Cancer Biology and Immunology, Amsterdam, Netherlands; 3 Amsterdam Institute for Immunology and Infectious Diseases, Cancer Immunology, Amsterdam, Netherlands; 4 Amsterdam UMC, Location Vrije Universiteit Amsterdam, Medical Oncology, Amsterdam, Netherlands; 5 Cancer Center Amsterdam, Imaging and Biomarkers, Amsterdam, Netherlands

**Keywords:** Immune cell attraction, Tumor secretome, Chemokines, Dendritic cells, Head and neck squamous cell carcinoma, Cancer-associated fibroblasts

## Abstract

Immune checkpoint inhibitors are approved for recurrent/metastatic head and neck squamous cell carcinoma (HNSCC) but the response rate is only 13–18%. For an effective antitumor immune response, trafficking of immune cells to the tumor microenvironment (TME) is essential. We aimed to better understand immune cell migration as well as the involved chemokines in HNSCC. A transwell assay was used to study immune cell migration toward TME-conditioned medium. While T cell migration was not observed, conventional dendritic cell (cDC) migration was induced by TME-conditioned media. cDC migration correlated with various proteins in the TME secretome. CCL8, CXCL5, CCL13 and CCL7 were tested in validation experiments and addition of these chemokines induced cDC migration. Using single cell RNA-sequencing, we observed expression of CCL8, CXCL5, CCL13 and CCL7 in cancer-associated fibroblasts (CAFs). Depleting fibroblasts led to reduced cDC migration. Thus CAFs, while often seen as suppressors of antitumor immunity, play a role in attracting cDCs toward the head and neck cancer TME, which might be crucial for effective antitumor immunity and response to therapies. Indeed, we found RNA expression signatures of the indicated chemokines, cDC and CAF subpopulations, to be significantly higher in baseline tumor specimen of patients with a major pathological response to pre-surgical anti-PD-1 treatment compared to non-responding patients.

**Supplementary Information:**

The online version contains supplementary material available at 10.1007/s00262-024-03809-z.

## Introduction

Head and neck squamous cell carcinoma (HNSCC) is the seventh most common cancer worldwide with 840,000 new cases and 450,000 deaths in 2020 and has a recurrence rate of 50% [1–3]. HNSCCs are most frequently located in the mucosal linings of the oral cavity, hypopharynx, larynx or oropharynx. Tumors of the oral cavity, hypopharynx and larynx are predominantly carcinogen-driven. Oropharyngeal SCCs (OPSCC) can be divided into tumors induced by persistent human papillomavirus (HPV) infection indicated as HPV-positive OPSCC, and HPV-negative OPSCC that follow the standard etiology, which are seen as separate disease entities due to divergent etiology, biology and clinical presentation. Patients with HPV-positive OPSCCs have an improved prognosis over patients with HPV-negative tumors [4–6]. Treatment regimens for HNSCCs differ per anatomical site and tumor stage, and include surgery, radiotherapy and/or chemoradiotherapy [6, 7]. In addition to above-mentioned treatment options, recurrent and metastatic HNSCC can be treated with immune checkpoint inhibitors (ICI) directed against programmed cell death protein-1 (PD-1). However, only 13–18% of the patients achieve a durable response [8, 9]. In the preoperative setting for patients with resectable, locally-advanced disease, clinical trials with anti-PD-(L)1 monotherapy have reported pathological response rates of on average 9.7% [10]. In order to improve this response rate to ICI for patients with HNSCCs, there is a need to better understand the tumor microenvironment (TME).

The TME comprises a number of cellular elements such as immune cells, endothelial cells, pericytes and stromal cells, as well as non-cellular elements such as the extracellular matrix (ECM) and proteins in the secretome secreted by cells from the TME [11]. Cancer-associated fibroblasts (CAFs) are part of the tumor stroma and are known for their plasticity and heterogeneity. While no consensus has been reached yet, the two most prevailing classes of CAFs are myofibroblastic CAFs (myCAFs) and inflammatory CAFs (iCAFs). myCAFs are known to produce ECM, have contractile properties as result of α-smooth muscle actin (αSMA) expression, promote tumor invasiveness and hamper immune infiltration into the TME. iCAFs produce cytokines like interleukin (IL)-6 and C-X-C motif chemokine ligand 12 (CXCL12), which recruit myeloid-derived suppressor cells (MDSCs) and M2-like macrophages and are known for their immunosuppressive features [12, 13].

Dendritic cells (DCs), part of the myeloid lineage of the immune system, play a central role in the immune response against cancer as they form a bridge between the innate and the adaptive immunity. DCs can be categorized into plasmacytoid DCs (pDCs), monocyte-derived DCs (moDCs) and conventional DCs (cDCs) with the latter being further divided into cDC type 1 (cDC1) and cDC type 2 (cDC2). In recent years, DCs are not only appreciated for their importance in initiating an effective antitumor T cell response in the tdLN, but also in retaining this at the tumor site. Specifically, DCs in the TME induce expansion of memory and effector T cells [13]. Moreover, it is thought that absence of DCs in immune-desert tumors might underlie the lack of T cells [13]. Of note, constant trafficking of DCs and T cells between the tumor and tdLN is required for tumor control [14–16]. The capacity of immune cells to infiltrate the TME depends on various factors, such as ECM, nutrient gradients, hypoxia, acidity and chemokines [13]. A comprehensive understanding of immune cell migration toward the TME of head and neck cancers is lacking.

Here we investigated immune cell migration toward the secretome of head and neck cancers. We employed an in vitro transwell migration assay with HNSCC-derived cell lines as attractants, as well as TME-conditioned media obtained from overnight cultures of enzyme-digested head and neck tumors from various anatomical sites. With this study, we aimed to gain a better understanding of the TME of head and neck cancer including their secretome, to identify the cells recruited, the chemokines responsible for recruiting these immune cells, and the cells within the TME that might be involved in the recruitment. Finally, we linked our in vitro findings to the pathological response to anti-PD-1 immunotherapy in a cohort of patients with oral cavity SCC (OCSCC) within the NeoNivo clinical trial (NCT 03843515) [17].

## Materials and methods

### Transwell migration assay with cell lines

HNSCC cell lines (Supplementary Table 1) were cultured in Dulbecco’s Modified Eagle Medium (DMEM, Gibco) containing 4.5 g/L D-Glucose, 4 mM L-Glutamine and 25 mM HEPES. This medium was supplemented with heat-inactivated fetal bovine serum (FBS, Biological Industries) and 1 mM sodium pyruvate, referred to as ‘DMEM-complete’. Cell lines were cultured at 37 °C and 5% CO_2_. Two days prior to the transwell migration assay, cell lines were seeded in 500 µl DMEM-complete to a confluency of 70% on the day of the experiment. 24-well plates with 5µm pore polycarbonate transwell inserts (Corning) were used. One day before the experiment, medium was replaced with DMEM containing 4.5 g/L D-Glucose, 4 mM L-Glutamine, 25 mM HEPES, 1 mM sodium pyruvate and 0.5% bovine serum albumin (BSA), referred to as ‘serum-free DMEM’. For the transwell migration assay, serum-free DMEM and a chemokine mix (Table 1) were used as negative and positive control, respectively.

**Table 1:** Chemokine mix as positive control in transwell migration assays

Chemokine	Concentration	Manufacturer
CCL2	50 ng/ml	Miltenyi
CCL4	50 ng/ml	PeproTech
CCL19	1000 ng/ml	Miltenyi
CCL20	100 ng/ml	PeproTech
CXCL10	500 ng/ml	PeproTech
CXCL12	100 ng/ml	PeproTech
CXCL13	1000 ng/ml	Miltenyi
GM-CSF	500 ng/ml	Miltenyi

CC motif chemokine ligand (CCL), C-X-C motif chemokine ligand (CXCL), granulocyte–macrophage colony-stimulating factor (GM-CSF)

Human peripheral blood mononuclear cells (PBMCs) were isolated from blood of healthy donors. 5 × 10^5^ PBMCs, in 100µl serum-free DMEM, were loaded into the upper compartment. The 24-well plate was incubated at 37 °C and 5% CO_2_. After six hours, the inserts were removed. From a control well to which no PBMC were added, 30 µl of the conditioned medium from each HNSCC cell line was stored at − 20 °C until further use. All cells from the lower wells were harvested. 100 µl 1 mM ethylenediaminetetraacetic acid (EDTA) in phosphate-buffered saline (PBS) was added and incubated at 37 °C for 15 min to detach the remaining cells. Migrated cells were quantified using flow cytometry.

### Transwell migration assay with TME-conditioned medium

Within 24 h of surgical removal, treatment-naive tumor biopsies were dissociated as reported previously [18]. In total, 1 × 10^5^ cells in 100µl Roswell Park Memorial Institute (RPMI) 1640 medium (Lonza) supplemented with 10% FBS, penicillin, streptomycin and L-glutamine (pen/strep/glut) were incubated at 37 °C and 5% CO_2_. After 24 h, 90 µl conditioned medium was collected, centrifuged for 5 min at 300 g and 80 µl of the TME-conditioned medium was stored at − 20 °C until further use.

For the transwell migration assay, 80 µl TME-conditioned medium was thawed, 10 µl was used for secretome analysis and 70 µl was diluted in 400 µl serum-free DMEM. 200 µl diluted TME-conditioned medium was plated twice (for experimental replicates) in a 96-well plate with 5 µm pore size inserts (Corning). 4 × 10^5^ human PBMCs in 80 µl serum-free DMEM were added in the inserts. For experimental replicates, two PBMC donors per TME-conditioned medium were included. Serum-free DMEM and a chemokine mix were used as negative and positive controls. Both contained the same percentage RPMI with 10% FBS, pen/strep/glut as the diluted TME-conditioned media. After 6 h of incubation at 37 °C, 5% CO_2_, cells from the lower compartment were harvested and quantified using flow cytometry.

### Transwell migration assay with chemokines

For the transwell migration assay with chemokines, a similar workflow was followed as described in Transwell migration assay with cell lines, except that no cells were plated in the lower compartment, which instead contained chemokine conditions as outlined in Table 2.

**Table 2:** Conditions for transwell migration assay with chemokines

Condition	Concentration	Manufacturer
CCL7 (MCP-3)	2 µg/mL	PeproTech
CCL8 (MCP-2)	2 µg/mL	PeproTech
CCL13 (MCP-4)	2 µg/mL	PeproTech
CXCL5 (ENA-78)	2 µg/mL	PeproTech
CCL7, CCL8, CCL13 and CXCL5	500 ng/mL	PeproTech
CCL7, CCL8, CCL13 and CXCL5	1 µg/mL	PeproTech
CCL7, CCL8, CCL13 and CXCL5	2 µg/mL	PeproTech

CC motif chemokine ligand (CCL), C-X-C motif chemokine ligand (CXCL), Monocyte chemoattractant protein (MCP), epithelial neutrophil-activating protein 78 (ENA-78). Chemokine dilutions were made in serum-free DMEM

### Transwell migration assay after fibroblast depletion

Following tumor dissociation, fibroblast depletion was performed using magnetic-activated cell sorting (MACS). In brief, freshly dissociated single cells were resuspended in PBS supplemented with 0.5% BSA and 2mM EDTA and incubated with 20 µl Anti-Fibroblast MicroBeads, human (Miltenyi Biotec) per 10^7^ cells according to manufacturer instructions. After 30 min of incubation at room temperature, cells were washed with PBS supplemented with 0.5% BSA and 2mM EDTA and added to a pre-wetted magnetic MS column (Miltenyi Biotec) including 70µm pre-separation filter (Miltenyi Biotec). The fraction depleted of fibroblasts (flow through) was collected in RPMI supplemented with 10% FBS and pen/strep/glut, and 1 × 10^5^ cells in 100µl were incubated at 37 °C and 5% CO_2_. After 24 h, 90µl conditioned medium was collected, centrifuged for 5 min at 300 g and 80 µl of the conditioned medium was stored at − 20 °C until further use. Besides overnight cultures, cells were stained for phenotyping using flow cytometry. For the transwell experiment, the same workflow was used as described for the transwell migration assay with TME-conditioned medium.

### Flow cytometry

After harvesting the cells from the lower compartments of the transwell migration assays, they were centrifuged at 300 g for 10 min at 4 °C, resuspended in 70 µl PBS supplemented with 0.1% BSA and 0.02% NaN_3_ (FACS-buffer) and incubated for 30 min at 4 °C with the fluorophore-coupled, murine anti-human monoclonal antibodies (mAbs) listed in Table 3. After incubation, the cells were washed and resuspended in 300 µl PBS supplemented with 0.1% BSA and 0.02% NaN_3_. 20 µl of 123 count eBeads (Thermo Fisher Scientific) were added for quantification.

**Table 3:** Fluorophore-coupled monoclonal antibodies (mAbs) used for flow cytometry staining

Assay	mAbs	Dilution	Manufacturer
Transwell migration	CD45-AF700	1:200	Biolegend
	CD11c-APC	1:100	BD Biosciences
	CD14-PerCP-Cy5.5	1:20	BD Biosciences
	CD123- BV650	1:30	BD Biosciences
	CD3-BV510	1:50	Biosciences
	CD19-BV605	1:50	BD Biosciences
	CD141-BV711	1:50	BD Biosciences
	CD1c-PE-Cy7	1:100	Sony
	CD4-BV786	1:50	BD Biosciences
	CD8-PE-CF594	1:200	BD Biosciences
	CD45RA-APC-H7	1:100	BD Biosciences
	CD127-BV421	1:50	BD Biosciences
	CD25-PE	1:50	BD Biosciences
	BDCA2-FITC	1:20	Miltenyi
Fibroblast depletion	CD45-AF700	1:200	Biolegend
	Podoplanin-PE	1:30	Biolegend
	PDGFRα-APC	1:30	Biolegend
	EpCam-BV421	1:30	Biosciences
Chemokine receptor staining	CXCR2-FITC	1:20	Biolegend
	CCR2-BV421	1:40	Biolegend
	CCR5-BV786	1:100	Biolegend
	CD25-PE	1:50	BD Biosciences
	CD14-PerCP-Cy5.5	1:20	BD Biosciences
	CD8-PE-CF594	1:200	BD Biosciences
	CD1c-PE-Cy7	1:100	Sony
	CD11c-APC	1:100	BD Biosciences
	CD45-AF700	1:200	Biolegend
	CD45RA-APC-H7	1:100	BD Biosciences
	CD3-BV510	1:50	BD Biosciences
	CD19-BV605	1:50	BD Biosciences
	CD123-BV650	1:30	BD Biosciences
	CD141-BV711	1:50	BD Biosciences

Blood dendritic cell antigen 2 (BDCA2), platelet derived growth factor receptor α (PDGFRα), epithelial cell adhesion molecule (EpCam), C-X-C motif chemokine receptor (CXCR), C–C chemokine receptor (CCR)

Following the fibroblast depletion using MACS, cells were resuspended in FACS-buffer and incubated for 30 min at 4 °C with mAbs specified in Table 3. For the chemokine receptor staining, PBMCs from healthy donors were resuspended in FACS-buffer and incubated for 30 min at 4 °C with mAbs listed in Table 3. After measurement using the BD LSR Fortessa X-20 cell analyzer, data were analyzed with FCS Express 6. See Supplementary Fig. 1 for gating strategy.

### Proximity extension protein assay

Collected conditioned media from cell lines as well as from dissociated tumor samples was cultured for 24 h, stored at − 20 °C and sent to Arcadia (UMC Utrecht) for secretome analysis using an Olink Target 96 proximity extension assay, as previously described [19, 20]. Normalized protein expression (NPX) values were measured for 92 proteins (Supplementary Table 2).

### Single cell RNA-sequencing

Following tumor dissociation, single cell RNA-sequencing (scRNA-seq) was performed for nine HNSCC specimens, as previously described [18]. In addition, scRNA-seq data from six OCSCC specimens were downloaded [21] and analyzed using the same steps and settings as previously described for our own dataset [18].

### Gene signature analysis on immunotherapy-treated oral cavity SCC

Gene set signature analysis was performed on RNA-seq data obtained from a previously published cohort of anti-PD-1 immunotherapy-treated OCSCC patients [17]. In short, sixteen treatment-naïve patients were treated with a single neoadjuvant dose of 480 mg nivolumab prior to major surgery with curative intent. Tumor specimens were obtained at baseline from tumor biopsies. Histopathologic evaluation of surgical specimens showed three patients with a major pathologic response (MPR), which were analyzed against the remainder of thirteen patients with no pathologic response (NPR). Sample workup for RNA-seq and data pipelines have been previously described [17]. Gene set signature scores for chemokines, general DCs, cDC1s (general DC + cDC1 specific), cDC2s (general DC + cDC2 specific), overall DCs (cDC1 + cDC2), myCAFs, iCAFs, elastic CAFs (eCAFs) and pericytes (Supplementary Table 3) were calculated for each sample as previously described [22].

### Statistical analysis

For the migration analyses, all absolute migration counts were normalized to migration toward the positive control (chemokine mix). Migration induction was defined as a mean cell count higher than the negative control (medium only) by at least two standard deviations (SD) of the negative control replicates. Per cell type, donors were excluded if the ratio of migration toward the positive and negative control was less than 2.5. Statistical analyses were executed using GraphPad Prism 9.3.1 software or R version 4.2.3. Paired nonparametric Wilcoxon signed-rank and unpaired nonparametric Mann–Whitney tests were used to compare two paired or unpaired groups, respectively. Unpaired nonparametric Kruskal–Wallis and paired nonparametric Friedman tests with Dunn’s tests to obtain *p*-values from multiple comparisons were used to compare multiple paired or unpaired groups, respectively. *p* < 0.05 was considered as statistically significant.

Some figures were generated using Servier Medical Art, licensed under a Creative Commons Attribution 3.0 Unported License.

## Results

### Immune cell migration induced by HNSCC cell lines and TME-conditioned medium

**Fig. 1:**
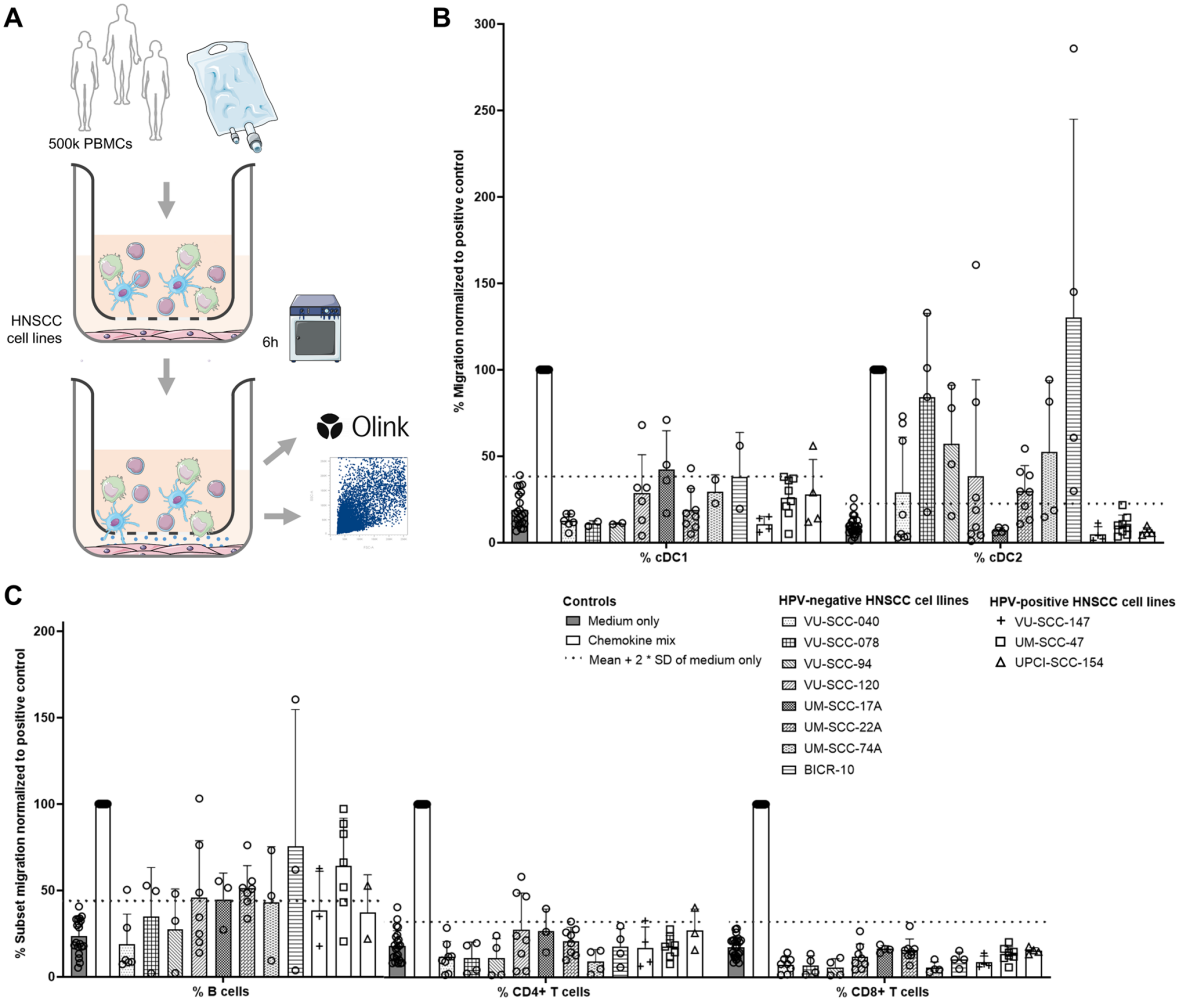
Migration of immune subsets toward head and neck cancer cell lines. **A** Schematic overview of transwell migration assay workflow. **B**, **C** Migration of, **B** conventional dendritic cells type 1 (cDC1s), conventional dendritic cell type 2 (cDC2s), **C** B cells, CD4 + T cells and CD8 + T cells (x-axis) toward human papillomavirus (HPV)-negative and -positive HNSCC cell lines (see legend). On the x-axis the various cell lines and type of migrated cells are depicted. On the y-axis the percentage of migrated cells normalized to the number of migrated cells in the positive control chemokine mix is depicted. Symbols represent experimental replicates using various PBMC donors. Dotted line indicates the cut-off represented by the mean plus two times the standard deviation (SD) of migration toward the negative control (medium only). Data are presented as mean and error bars indicate standard deviations. When the mean exceeds the dotted line, it is considered as active migration events

Using a transwell migration assay, we examined whether, and which immune cells migrated toward HNSCC cell lines (Fig. 1). While cDC2 and B cell migration was observed toward about half of the cell lines (seven and five out of eleven, respectively), cDC1 migration was only observed for one out of eleven (UM-SCC-17A) (Fig. 1B). Evidently, CD4 + and CD8 + T cells did not migrate toward any of these HNSCC cell lines (Fig. 1C).

Whereas for cDC1s, B cells and T cells no difference was observed between migration toward HPV-negative and -positive cell lines, more cDC2s migrated toward HPV-negative cell lines (Fig. 1B, Supplementary Fig.2). Specifically, migration of cDC2s was observed toward seven out of eight HPV-negative cell lines while no migration occurred toward three HPV-positive cell lines (*p* = 0.02 by Fisher’s exact test).

**Fig. 2:**
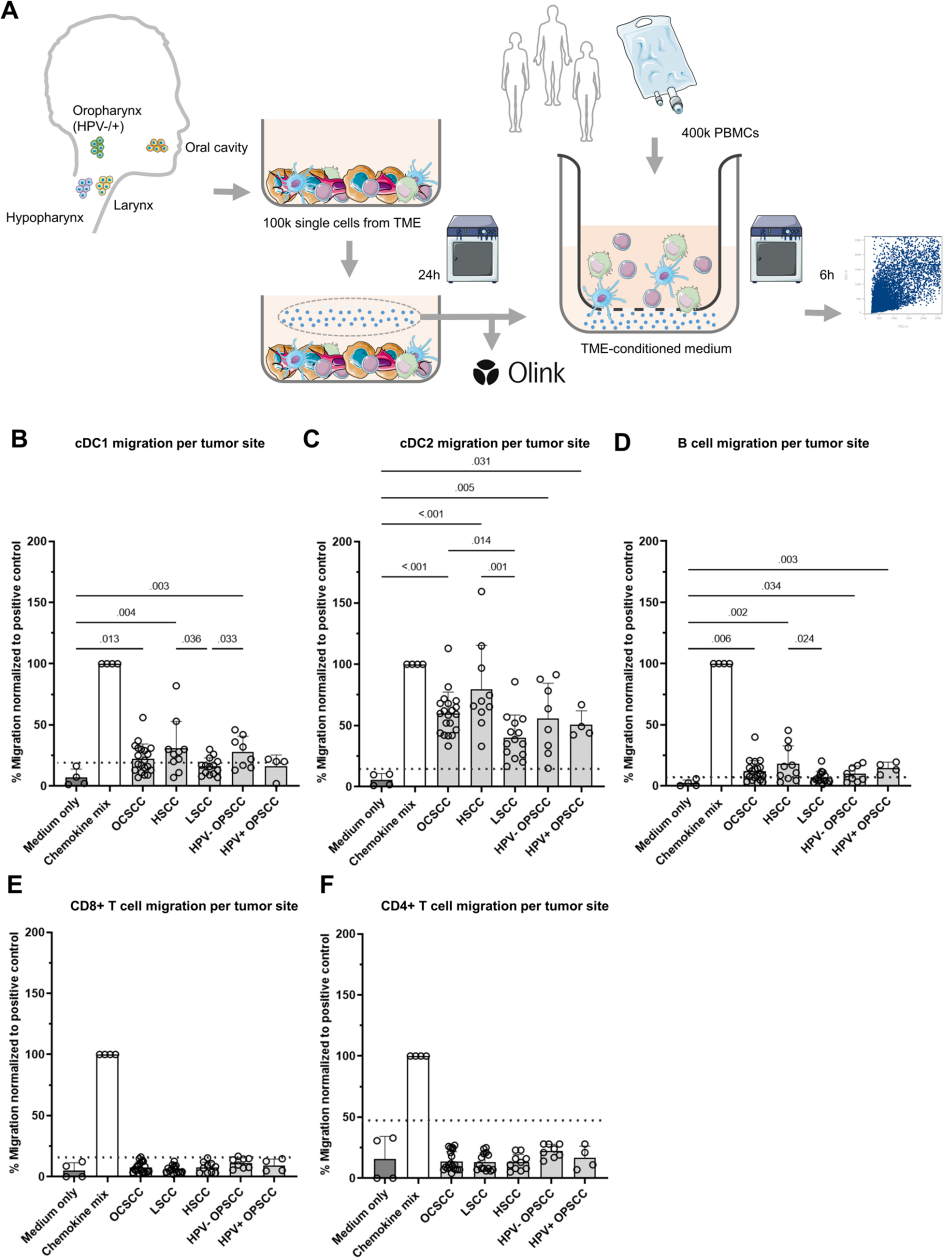
Migration of immune subsets toward TME-conditioned media. **A** Schematic overview of workflow for transwell migration assay using tumor microenvironment, (TME)-conditioned medium. **B**–**F** Migration of **B** conventional dendritic cells type 1 (cDC1), **C** conventional dendritic cell type 2 (cDC2), **D** B cells, **E** CD8 + T cells and **F** CD4 + T cells (y-axis) toward TME-conditioned medium from various anatomical sites (x-axis), normalized to migration to the chemokine mix as positive control. Symbols represent experimental replicates from various human PBMC donors. Each conditioned medium sample was split in two to obtain replicates. Dotted line indicates the mean plus two times the standard deviation (SD) of migration toward negative control (medium only). Data are presented as mean and error bars indicate standard deviations. When the average exceeds the dotted line, it is considered as active migration. An unpaired nonparametric Kruskal–Wallis test and a Dunn’s test was performed to obtain *p*-values, which were included in the figure in case *p* < 0.05. OCSCC, oral cavity squamous cell carcinoma; LSCC, laryngeal squamous cell carcinoma; HSCC, hypopharyngeal squamous cell carcinoma; HPV, human papillomavirus; OPSCC, oropharyngeal squamous cell carcinoma

Cultured cancer cell lines miss the complexity of the TME present in tumors, and may therefore lack the ability to attract specific immune cell populations. Hence, we next investigated immune cell migration toward TME-conditioned media, originating from biopsies obtained from various HNSCC anatomical sites, including HPV-negative and -positive OPSCCs (Fig. 2A, Supplementary Table 4). Interestingly, while T cells did not migrate toward TME-conditioned media, DCs and B cells were recruited by conditioned medium from most tumor specimens. Specifically, cDC1s and B cells migrated toward the majority, and cDC2s migrated toward all TME-conditioned media (Supplementary Fig. 3). In general, laryngeal SCC (LSCC)-conditioned media attracted fewer immune cell populations compared to OCSCC, hypopharyngeal SCC (HSCC) and HPV-negative OPSCC (Fig. 2B–D).

### Chemokines in TME-derived secretomes associated with migration of dendritic cells

**Fig. 3:**
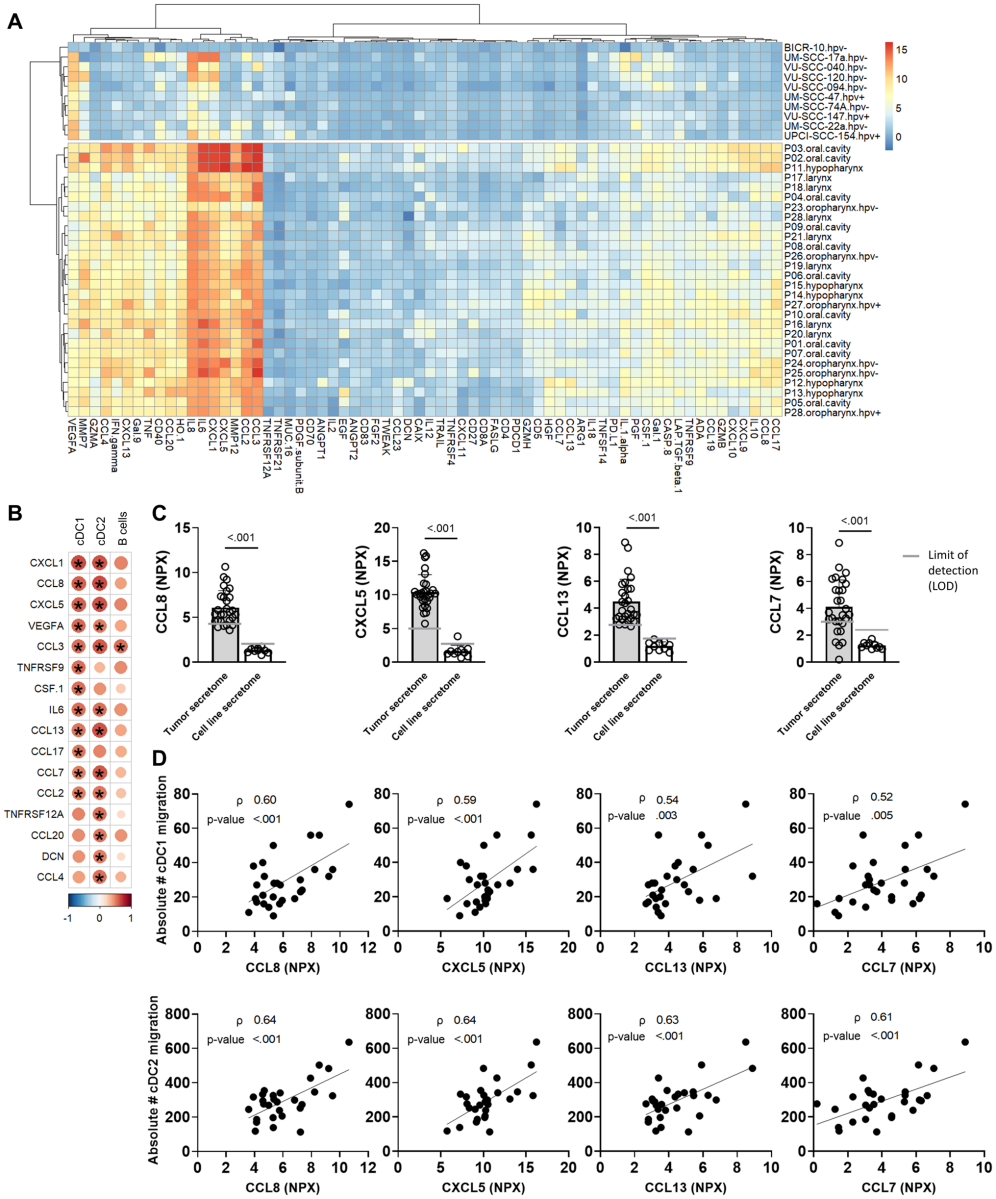
Secretome analysis of conditioned medium derived from head and neck cancer cell lines and from tumor biopsies. **A** Unsupervised hierarchical clustering of secretome normalized protein expression (NPX) (x-axis) from head and neck squamous cell carcinoma (HNSCC) cell lines and HNSCC biopsies (y-axis). **B** Pearson correlation (using Benjamini–Hochberg false discovery rate correction) between migration of conventional type 1 dendritic cells (cDC1s), conventional type 2 dendritic cells (cDC2s) and B cells toward tumor microenvironment (TME)-conditioned medium (in absolute cell counts) and secretome protein levels (in NPX). Colors indicate correlation coefficient, * indicates *p* < 0.05. Proteins either significantly correlating with cDC1, cDC2 or B cell migration are displayed, and ordered based on highest to lowest cDC1 migration correlation coefficient. **C** Out of twelve proteins which significantly correlated with cDC1 migration, CCL8, CXCL5, CCL13 and CCL7 were below the limit of detection (LOD) in cell line-derived secretomes (white bars) while present in the majority of TME-derived secretomes (gray bars). *P*-values obtained by Mann–Whitney test and were included in the figure in case *p* < 0.05. **D** Pearson correlation between CCL8, CXCL5, CCL13 and CCL7 protein levels (in NPX value, x-axis) and cDC1, cDC2 migration in absolute counts (#, y-axis). *P*-values and Pearson’s correlation coefficients (*ρ*) are depicted in the graphs

To identify chemokines that might be relevant for immune cell attraction in the TME of HNSCC, we examined the secretome of HNSCC cell lines and overnight cultures of tumor biopsies by proximity extension assay (Fig. 3A). Evidently, proteins were found at higher levels in secretomes from tumors compared to those from tumor cell lines. This suggests that those proteins were likely derived from the stromal and/or immune infiltrate compartment, although we cannot formally exclude that tumor cells reduce secretion of chemokines after prolonged culturing.

As migration toward TME-conditioned media was solely observed for DCs and B cells, we examined whether we could find correlations between their migration and protein levels within the TME-derived secretome (Fig. 3B). Of the 65 proteins detected within the secretome of tumors, sixteen proteins correlated with either cDC1 or cDC2 migration and only one correlated with B cell migration (CCL3). For further selection of chemokines for validation, we focused on chemokines correlating with cDC1s; the levels of twelve proteins positively correlated with the migration of cDC1s (Fig. 3B). Since migration was more evident toward TME-derived secretomes compared to cell line secretomes, we compared protein levels between TME and cell lines. Four proteins absent in secretomes from cell lines were present in the majority of TME-derived secretomes (Fig. 3C), indicating that those might induce cDC1 migration. Those four proteins, CCL8/MCP-2, CXCL5/ENA-78, CCL13/MCP-4 and CCL7/MCP-3, showed a strong correlation with cDC1 as well as cDC2 migration (Fig. 3D). Interestingly, we observed increased levels of both CCL7 and CCL13 in the secretome of HSCC compared to the secretome of LSCC (Supplementary Fig. 4), corresponding with the increased DC migration observed.

**Fig. 4:**
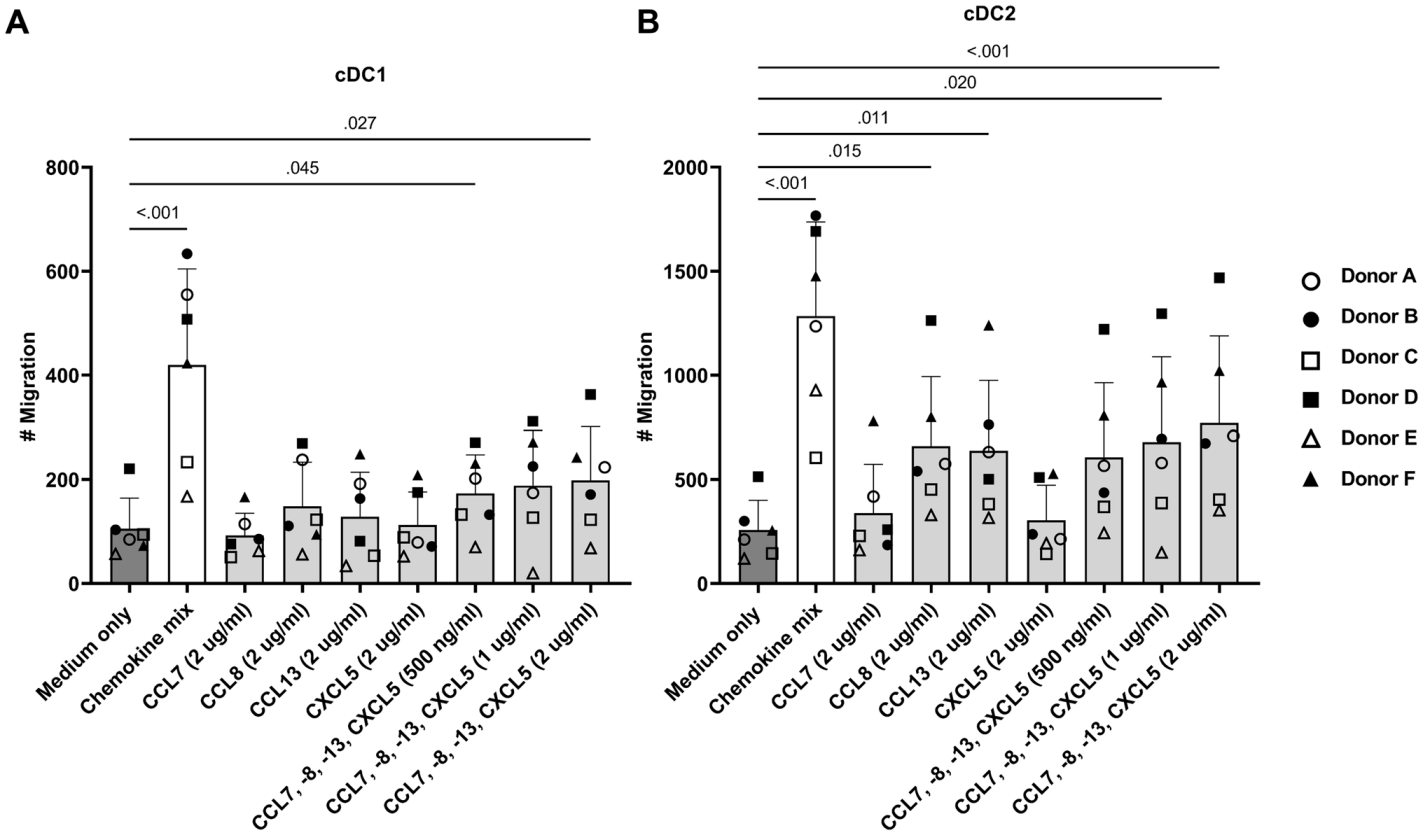
Chemokines CCL7, CCL8, CCL13 and CXCL5 induce dendritic cell migration. Transwell migration assay with chemokines CCL7, − 8, − 13 and CXCL5 (2 μg/ml) and the combination of the four chemokines (500 ng/ml, 1 μg/ml and 2 μg/ml) in the lower compartment (x-axis). Migration of **A** conventional dendritic cells type 1 (cDC1) and **B** conventional dendritic cells type 2 (cDC2), in absolute counts (#, y-axis) towards separate proteins or towards combination of proteins in increasing concentration. Symbols represent various PBMC donors. *P*-values were obtained by performing a paired non-parametric Friedman test with Dunn’s tests comparing all conditions with medium only and were included in the figure in case *p* < .05

To verify the role of CCL7, − 8, − 13 and CXCL5 in attracting DCs, we examined whether the chemokines by themselves or in combination could induce migration (Fig. 4). Indeed, the combination of the four chemokines induced both cDC1 and cDC2 migration. In addition, cDC2s were attracted by solely CCL8 or CCL13.

### Chemokine receptors CXCR2, CCR2 and CCR5 are present on dendritic cells

**Fig. 5:**
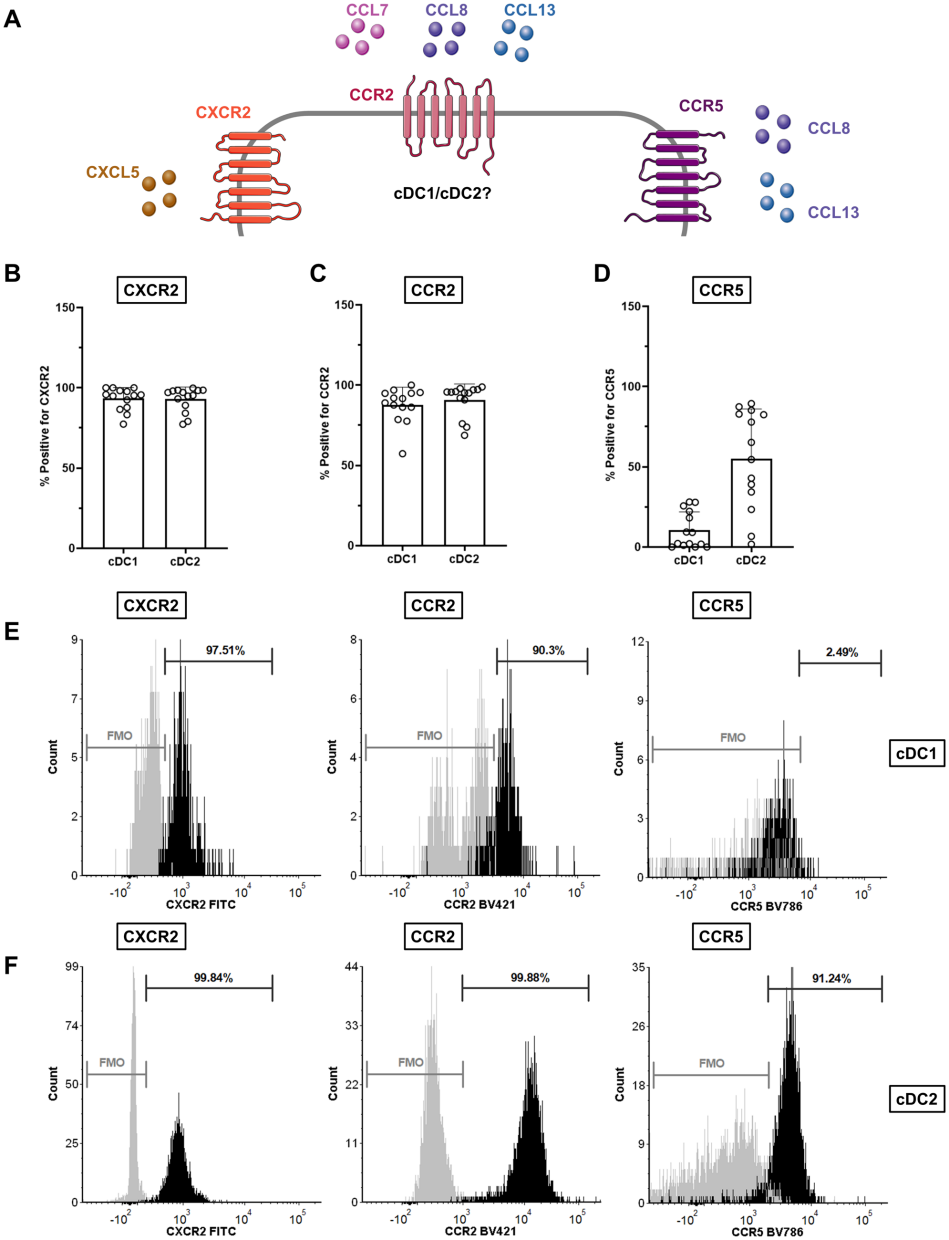
Chemokine receptor staining. **A** Schematic overview of chemokines and their receptors. **A**–**F** Peripheral blood mononuclear cells (PBMCs) from healthy donors were used to assess the fraction of conventional dendritic cells type 1 (cDC1) and conventional dendritic cells type 2 (cDC2) positive for chemokine receptors **B** CXCR2, **C** CCR2 and **D** CCR5 by immunostaining and flow cytometry analysis. *P*-values were obtained by a paired nonparametric Wilcoxon signed-rank test and were included in the figure in case *p* < 0.05. **E**–**F** Representative flow histograms of **E** cDC1s and **F** cDC2s with fluorescence minus one (FMO) in gray and full staining in black. Percentage of cells positive for chemokine receptors are indicated

As CXCL5 binds to chemokine receptor CXCR2, CCL7, CCL8 and CCL13 to CCR2, and CCL8 and CCL13 also to CCR5 [23, 24], we investigated the presence of those chemokine receptors on DCs (Fig. 5). CXCR2 and CCR2 were expressed by almost all DCs. CCR5 was only present on a fraction of the cDC1s while the majority of cDC2s expressed CCR5 (Fig. 5D). This could possibly explain the cDC2 migration toward CCL8 or CCL13 alone (Fig. 4B).

### Cancer-associated fibroblasts attract dendritic cells

**Fig. 6:**
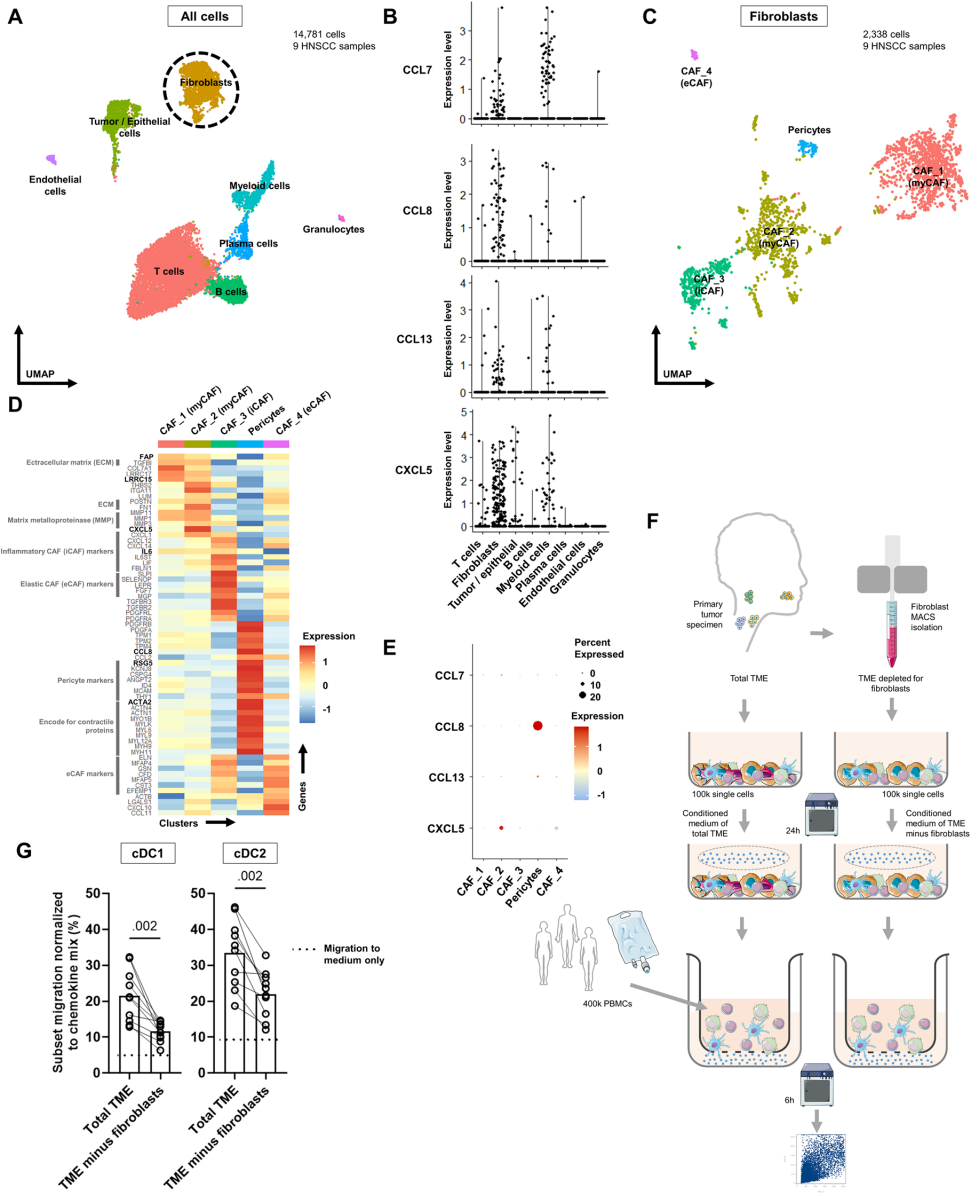
Fibroblasts responsible for attracting dendritic cells. **A** Uniform Manifold Approximation and Projection (UMAP) based on RNA expression of 14,781 cells from nine head and neck tumor specimens. **B** Expression levels (y-axis) of CCL7, -8, -13 and CXCL5 per cluster (x-axis). **C** Fibroblasts were isolated and subclustering was performed on 2,338 cells. 4 clusters of cancer-associated fibroblasts (CAFs) were discerned. CAF_1 and CAF_2 were annotated as myofibroblasts (myCAF), CAF_3 as inflammatory CAF (iCAF), CAF_4 elastic CAF (eCAF) and one cluster as pericytes. **D** Average expression level of marker genes (y-axis) per subcluster (x-axis). **E** Average expression of CCL7, -8, -13 and CXCL5 (y-axis) in each subcluster (x-axis). Size of the circles represents percentage of cells with positive expression of the involved chemokines. **F** Workflow of fibroblast MACS isolation from tumor specimen and transwell experiments using conditioned medium of total TME and from TME depleted of fibroblasts. **G** Migration of conventional dendritic cell type 1 (cDC1) and conventional dendritic cell type 2 (cDC2) normalized to the positive control chemokines mix (y-axis) toward conditioned medium derived either from the total TME or from the TME depleted of fibroblasts (x-axis). Dotted lined represents migration toward medium only. *P*-values were obtained by a paired nonparametric Wilcoxon signed-rank test and were included in case *p* < 0.05

To gain insight into which cells in the HNSCC TME could potentially secrete CCL7, -8, -13 and CXCL5, we performed analyses on our in-house scRNA-seq dataset of nine HNSCC specimens (Fig. 6, Supplementary Fig. 6, [17]). The highest expression of CCL8 was observed in the fibroblast cluster and expression of CCL7, CCL13 and CXCL5 was found in both fibroblasts as well as myeloid cells (Fig. 6B). This indicates that fibroblasts and myeloid cells might secrete CCL7, -8, -13 and CXCL5 in the TME and thereby attract DCs.

To examine which fibroblasts expressed the chemokines of interest, we performed subclustering (Fig. 6C). Four CAF clusters were discerned with expression of classical markers such as *FAP*, *VIM*, *PDGFRA* and *PDPN* (Fig. 6D). CAF_1 and CAF_2 were both annotated as myCAFs with *POSTN* and *TGFBI* expression (encoding ECM proteins) as well as *ACTA2* (αSMA), *MYH9* and *ACTN1* (encoding for contractile proteins). CAF_3 showed high expression of *IL6*, *CXCL1* and *CXCL12* with only minor *ACTA2* expression, and was annotated as iCAF [12]. Lastly, CAF_4 was annotated as eCAFs [25]. Of note, one small cluster was negative for most of the common CAF markers but did express pericyte markers such as *RGS5*, *MCAM* and high expression of *ACTA2* (Fig. 6D) [12, 26, 27]. Pericytes are vascular cells adjacent to endothelial cells and can also be a source of CAFs [28].

Only marginal *CCL7* expression was found in CAF_1, CAF_2 and CAF_3 (Fig. 6E). The highest *CCL8* expression was found in pericytes, with minimal expression in myCAFs. Likewise, *CCL13* was highest expressed by pericytes. Most potent *CXCL5* expression was observed in CAF_2. Summarizing, pericytes and cells from clusters CAF_1, CAF_2 (myCAFs) and CAF_3 (iCAFs) expressed *CCL7*, -*8*, -*13* and *CXCL5*, suggesting that those cells in the TME might secrete those proteins and consequently attract DCs. Comparable results were found in an external scRNA-seq dataset of six OCSCC (Supplementary Fig. 7) [21].

To validate this potential role for fibroblasts in attracting DCs, migration was measured toward TME-conditioned medium derived from tumor samples depleted of fibroblasts (Fig. 6F, Supplementary Fig. 8). As expected, migration of cDC1s and cDC2s was hindered in the fibroblast-depleted TME (Fig. 6G), suggesting that fibroblasts contribute to the attraction of DCs toward the TME of head and neck cancers.

### Gene set signature scores on immunotherapy-treated oral cancer SCC

**Fig. 7:**
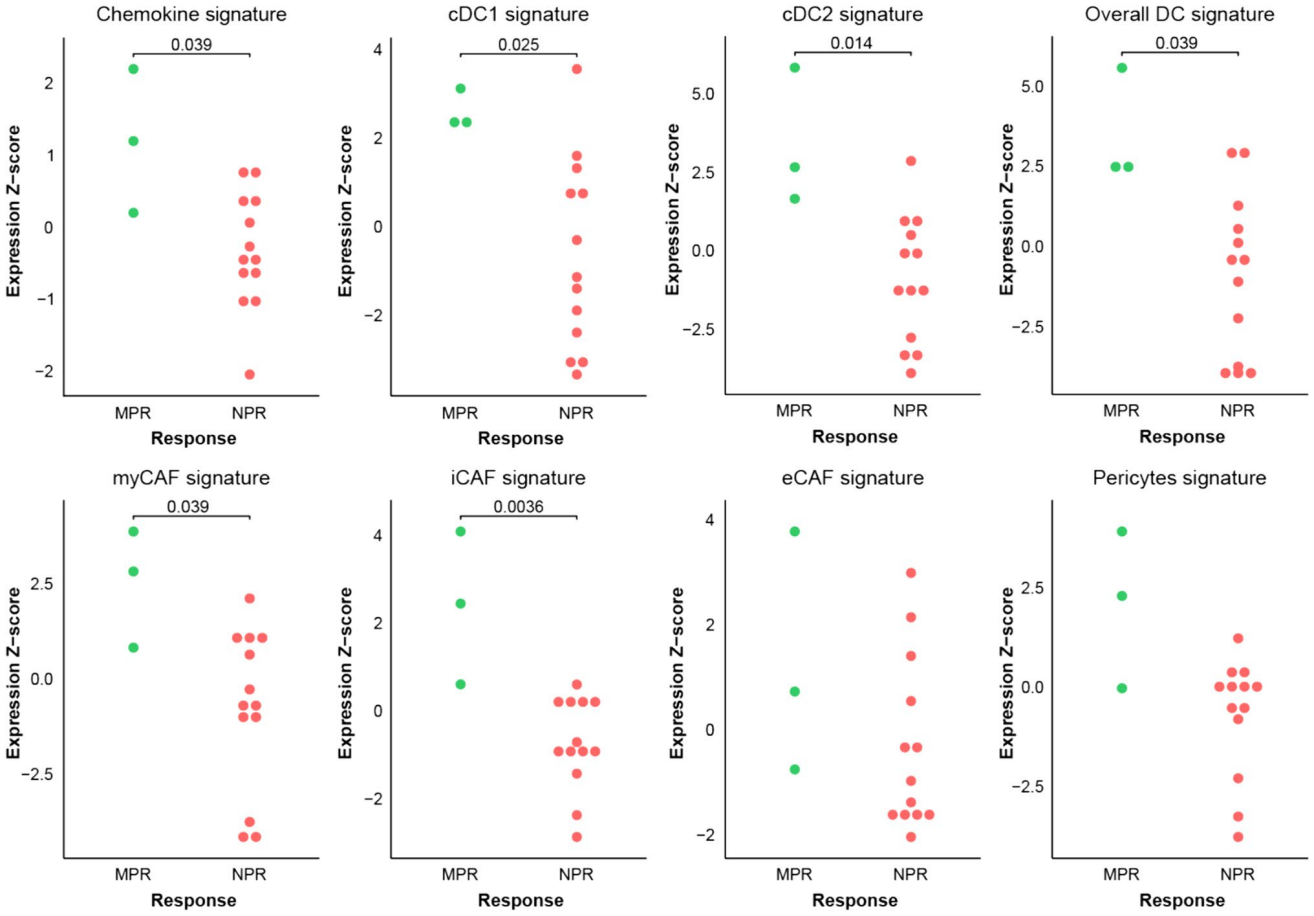
Gene set enrichment analysis on immunotherapy-treated cohort. Baseline expression Z-scores for each signature are depicted for responders (MPR; n = 3) vs non-responders (NPR; n = 13). *P*-values were obtained by a nonparametric Wilcoxon rank-sum test. MPR, major pathologic response; NPR, no pathologic response; cDC1, conventional dendritic cell type 1; cDC2, conventional dendritic cell type 2; myCAF, myofibroblastic CAF; iCAF, immunological CAF; eCAF, elastic CAF

We next assessed whether our in vitro observations could be linked to clinical response to ICI. For this we used an RNA-seq dataset from baseline tumor biopsies from patients included in the NeoNivo clinical trial, in which sixteen patients with locally-advanced OCSCC received one dose of the anti-PD-1 drug nivolumab prior to surgery [17]. We compared gene signatures for our chemokines (CCL7, -8, -13, CXCL5), cDCs and CAF populations between patients with a MPR after neoadjuvant treatment (*n* = 3) and patients without a pathological response (NPR) (*n* = 13). In line with our in vitro data, gene set signature analysis showed that responders had a significantly higher expression at baseline for the chemokine, cDC1, cDC2, overall DC (cDC1 + cDC2), myCAF and iCAF signatures, but not for the eCAF and pericyte signatures (Fig. 7).

## Discussion

In the current study we exploited a unique workflow to investigate immune cell migration toward HNSCC TME-conditioned medium. We found that DCs and B cells migrated toward TME-conditioned medium. Strong associations were found between DC migration and secretome levels of the chemokines CCL7, -8, -13 and CXCL5. mRNA expression of those chemokines was observed in fibroblasts using scRNA-seq and when depleting fibroblasts from the TME, fewer DCs were attracted. This indicates that CAFs contribute to the attraction of favorable immune infiltrates, such as DCs. CAFs have been described to secrete CCL7 in response to IL-1α from OCSCC cells [29, 30]. The effect of CCL7 on immune cell migration was not examined; however, CCL7 promoted OCSCC cell proliferation [29, 30], emphasizing that chemokines may exhibit a variety of functions. CCL8 and CXCL5 are generally known as pro-inflammatory cytokines, produced by M1-like macrophages [31, 32]. Conversely, Miyazaki et al*.* demonstrated that downregulating CXCL5 in an HNSCC cell line resulted in impaired migration of tumor cells [33], again underscoring the double-edged effects of certain chemokines. Of note, in our study marginal mRNA expression of *CXCL5* was observed in the epithelial/tumor cell cluster (Fig. 6B). It might be that CXCL5 is secreted by various cell types and that its effect varies depending on the location within the TME, the chemokine receptor and the cell type it engages. Along the same line, CCL13 has been described to be expressed by M2-like macrophages [31], which appears to induce OCSCC cell migration [34]. In our scRNA-seq analysis, we indeed observed several of these chemokines to be expressed by myeloid cells as well, and depletion of CAFs did not fully abrogate cDC migration (Fig. 6G), suggesting that these may also contribute to cDC attraction.

Our data indicate that CAFs are important for recruiting DCs. CAFs are mainly known for their pro-tumorigenic features, among which are the production of ECM, obstructing immune cells from entering the TME [13], and the attraction of suppressive immune cell populations [35]. Interestingly, Obradovic et al. [36] distinguished five CAF clusters and demonstrated that two of those clusters may predict ICI response and even enhance T cell cytotoxicity, while another CAF cluster was thought to induce immunosuppression, underlining that CAFs are remarkably heterogeneous [36]. In line with this, our study showed that CAFs contribute to DC recruitment. Moreover, gene set enrichment analysis in an immunotherapy-treated cohort of OCSCC patients showed that apart from a significantly higher baseline expression for all DC signatures, responders had a significantly higher baseline expression of myCAF and iCAF signatures. These findings strengthen the suggestion that these CAF subtypes might play pivotal roles in immunotherapy response through supporting the attracting of cDC populations.

T cell migration was neither observed toward HNSCC cell lines nor to TME-conditioned medium. The noted lack of T cell attraction may be one of the reasons why current treatments with anti-PD-1 are effective in only a minority of HNSCC patients. It would be valuable to understand which cytokines are responsible for stimulating or hampering the attraction of T cells into the TME of HNSCCs. The lack of T cell migration toward 28 TME-derived secretomes in itself is remarkable, given the presence of several well-known chemokines. First of all, CXCL9 and -10, generally known to be required for effector T cells trafficking into the tumor [13, 37, 38], were present in the majority of TME-derived secretomes. Secondly, Hoffmann et al*.* showed that CCL2/MCP-1, produced in HNSCC cell line-derived spheroids, induced CD2 + leukocyte infiltration in PBMC and spheroid co-cultures [39]. Of note, CCL2 was present in all TME-derived secretomes. These data strongly suggest that the absence of T cell trafficking may be caused by presence of inhibitory proteins or that the distance between T cells and chemokine-producing cells is of relevance. To illustrate the former, but likely not being the only factor, vascular endothelial growth factor (VEGF), present in all our TME-derived secretomes and produced by most HNSCC cell lines, is generally known to inhibit infiltration of T cells [13]. The secretome is like an orchestra; each protein has a separate role. However, the interplay and context of all together make the symphony and determine trafficking of immune cells.

While T cells were not attracted by TME-conditioned medium, we previously investigated the immune composition of HNSCC using flow cytometry and found T cells present in the TME [18]. Although the numbers differed per tumor, T cells were present in every tumor. Somewhat remarkable in the context of the current study, that suggests that the secretome of HNSCC is not able to recruit new T cells. This may simply reflect a timing issue, with T cells being recruited prior to and during tumor development, but recruitment being hampered within a fully-developed cancer. As the recruitment of recently primed T cells from the tdLN toward the TME is essential for a successful antitumor response [13], the lack of T cell migration might explain the marginal response rate of HNSCCs to ICIs.

We found less migration of DCs and B cells toward conditioned media derived from LSCC compared to other anatomical sites. Interestingly, low protein levels were found in the secretome of LSCC, particularly in comparison with HSCC-derived secretomes. We previously reported differences in the immune composition of HNSCCs at distinct sites [18, 19]. We did not find correlations between the fraction of B cells, T cells or myeloid cells quantified in the TME and migration of DCs and B cells toward the secretome of matched tumors. Thus, diminished migration toward LSCC-conditioned media is likely not explained by presence or absence of certain immune subsets within the TME. Whether the fraction of CAFs differed between the tumor specimen, and whether this may have contributed to different migration patterns, remains unknown since no markers to identify CAFs were included in the flow cytometry study [18].

Interestingly, we found a disparity in cDC2 migration between HPV-negative and HPV-positive HNSCC cell lines. While cDC2s migrated toward the majority (seven out of eight) of HPV-negative cell lines, no cDC2 migration was observed toward HPV-positive cell lines. Interestingly, no difference in migration was notable between HPV-negative and HPV-positive TME-conditioned media. The latter includes next to tumor cells also other cells such as immune and stromal cells. Therefore, we hypothesize that this difference should be a tumor intrinsic effect. In an attempt to understand this tumor intrinsic mechanism, we compared the secretome between HPV-negative and HPV-positive cell lines. The only apparent difference between their secretome profiles was higher levels of Galectin-9 (Gal-9) in HPV-positive compared to HPV-negative cell lines (Supplementary Fig. 5). Also, a negative correlation was found between cDC2 migration and levels of Gal-9. Indicating that Gal-9 in HPV-positive cell lines might have hampered cDC2 migration. Gal-9 has been described to be upregulated in multiple cancer types, with diverse effects [40, 41]. Interestingly, while Gal-9 levels were evidently higher in the secretome derived from the TME compared to cell lines, cDC2 migration was still observed toward all TME-conditioned media. As such, Gal-9 levels did not negatively correlate with cDC2 migration for the HNSCC biopsies. This might be explained by the overall higher levels of other proteins in the TME-derived secretome. By way of illustration, CCL7, -8, -13 and CXCL5, which positively correlated with cDC2 migration toward the TME-conditioned media, were all not present in the cell lines. The presence of these proteins might have overshadowed the suppressive effects of Gal-9.

Limitations of our study include PBMC donor variability which makes interpretations sometimes challenging. Some donors had to be excluded as they showed already quite some spontaneous cell migration. Also with this in vitro setting, we study the migration of peripheral immune cells from the blood toward the TME and could not mimic migration of T cells that are primed within the tdLN prior to migration to the TME. Moreover, the proximity extension protein Olink assay has a limitation, as protein levels could only be compared across samples but not between proteins. Finally, to examine the role of CAFs in attracting DCs, we depleted CAFs in general. It was technically infeasible to specifically deplete subpopulations of CAFs like myCAFs and/or pericytes.

## Conclusion

This work presents a unique, functional insight into immune cell migration toward HNSCC from different anatomical sites along the head and neck region. First of all, no T cell migration was observed toward either the cell lines or HNSCC secretome, possibly contributing to the low ICI response rate of HNSCC. Interestingly, immune cell migration toward, as well as protein levels in, the secretome differed across anatomical sites. CCL7, -8, -13 and CXCL5 in the TME secretome correlated strongly with cDC1 and cDC2 migration and together were able to attract cDC1 and cDC2. We noted expression of CCL7, -8, -13 and CXCL5 in fibroblasts with most expression of CXCL5 in myCAFs. When fibroblasts were depleted from the TME, DC migration was diminished, indicating that CAFs in HNSCCs are involved in attracting DCs. Lastly, cDC, myCAF and iCAF signatures were significantly higher in ICI responders compared to non-responders, highlighting their role in antitumor immunity.

## Supplementary Information

Below is the link to the electronic supplementary material.

Supplementary file1 (DOCX 1784 KB)

Supplementary file2 (XLSX 17 KB)

Supplementary file3 (XLSX 16 KB)

Supplementary file4 (XLSX 14 KB)

Supplementary file5 (XLSX 19 KB)

## Data Availability

Data are available upon reasonable request. Correspondence and request for materials should be addressed to Dr. Rieneke van de Ven. E-mail address: r.vandeven@amsterdamumc.nl Amsterdam UMC, location VUmc, De Boelelaan 1117—Zh 2A60, 1081 HV Amsterdam.

## Abbreviations

HNSCC: Head and neck squamous cell carcinoma
TME: Tumor microenvironment
scRNA-seq: Single cell RNA-sequencing
cDC: Conventional dendritic cell
CAF: Cancer-associated fibroblast
PD-1: Programmed cell death protein-1
