# Supplementary material for: Secretome and immune cell attraction analysis of head and neck cancers
Source: Cancer Immunol Immunother. 2024 Sep 9;73(11):229. doi: 10.1007/s00262-024-03809-z (PMC11383899; doi:10.1007/s00262-024-03809-z)
Supplement: Supplementary file 1 — Supplementary file1 (DOCX 1784 KB) [file 262_2024_3809_MOESM1_ESM.docx]

## Supplementary Figures


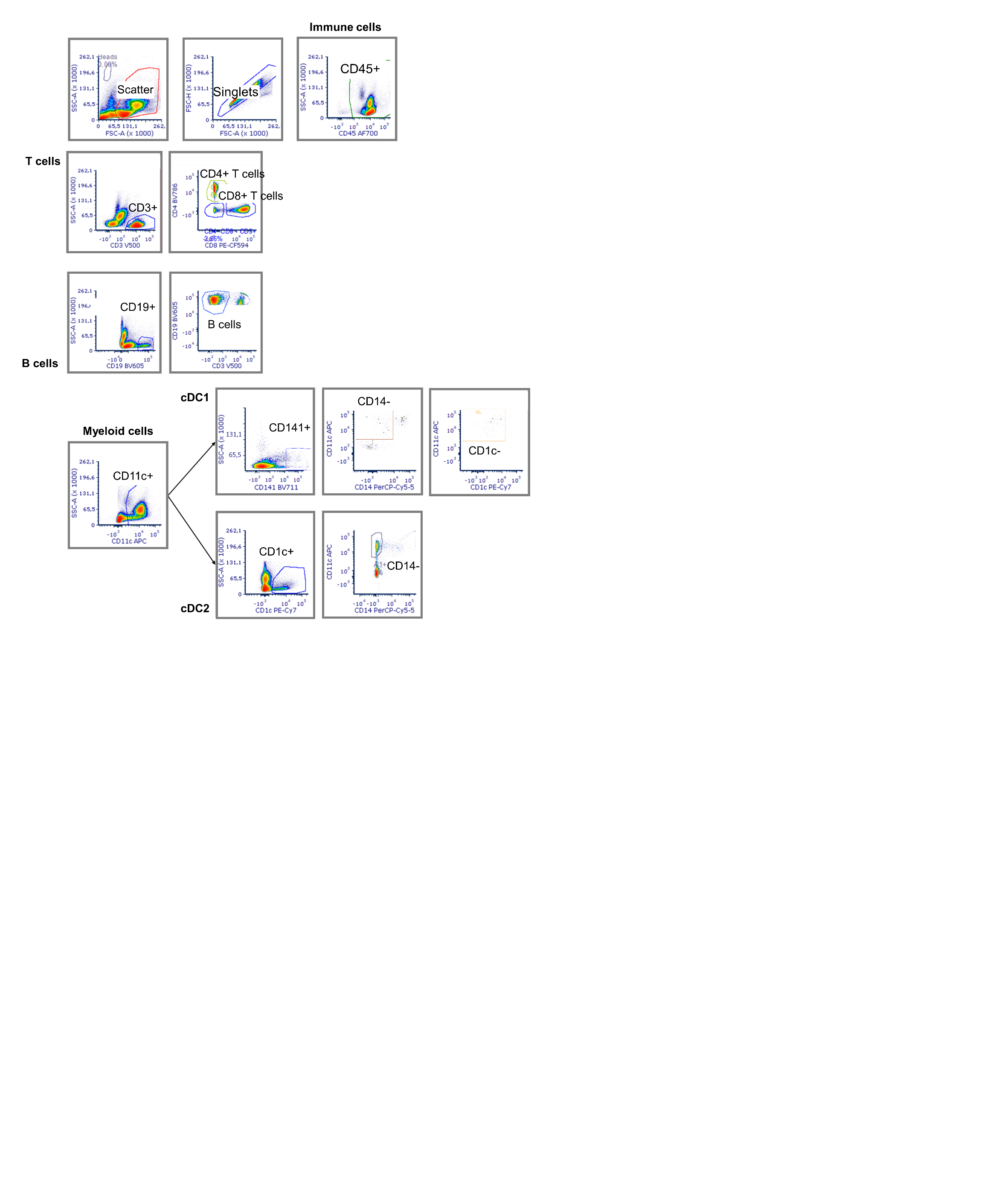


**Supplementary Fig 1. Gating strategy flow cytometric analysis following transwell migration assay**. Cells were gated using a scatter gate, followed by a singlet gate. Out of the CD45+ immune cells, CD3+ T cells (with CD4+ and CD8+ T cells), CD19+ B cells and CD11c+ myeloid cells were gated. For the conventional type 1 dendritic cells (cDC1s), CD141+, CD14-, CD1c- cells were gated. For the conventional type 2 dendritic cells (cDC2s), CD1c+, CD14- cells were gated.


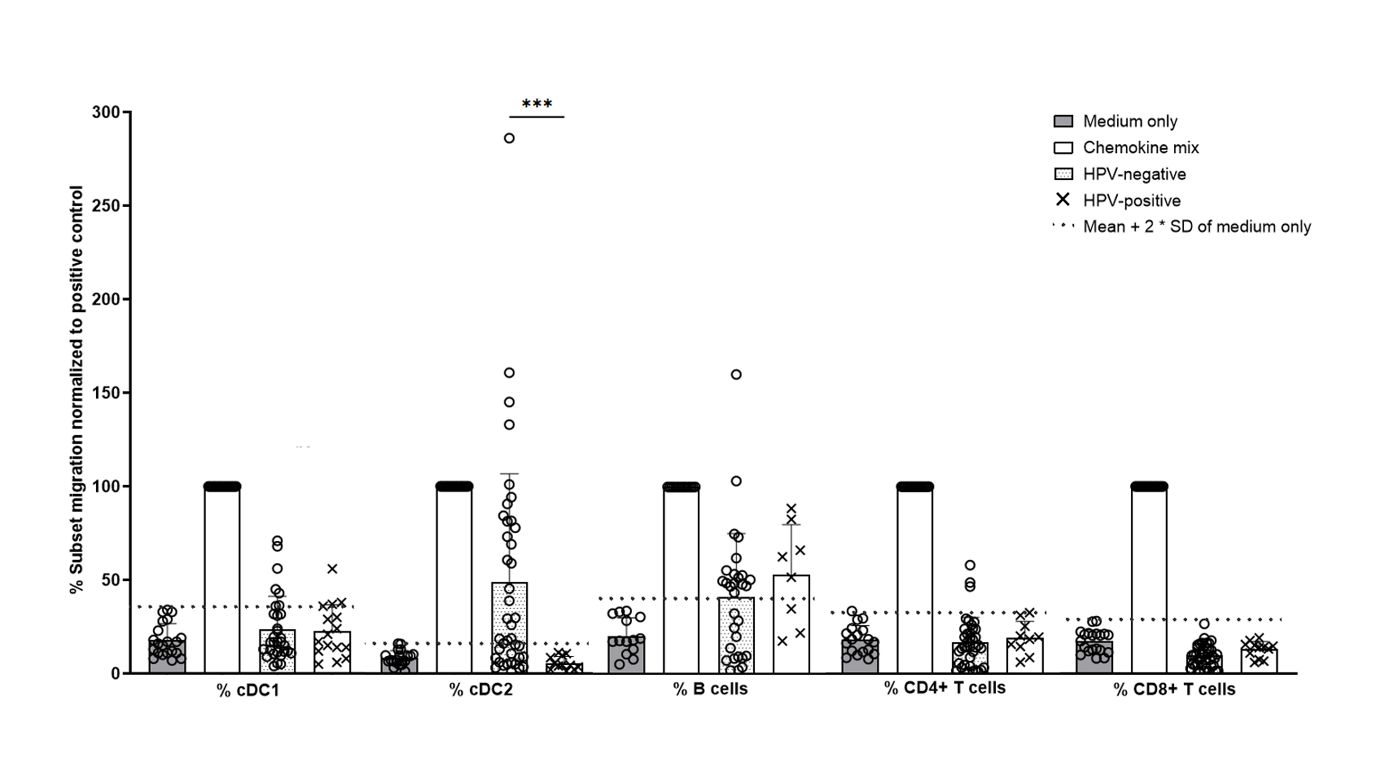


**Supplementary Fig 2. Migration of immune subsets towards human papillomavirus (HPV)-negative and -positive head and neck cancer cell lines.** Migration of conventional dendritic cells type 1 (cDC1), conventional dendritic cell type 2 (cDC2), B cells, CD4+ T cells and CD8+ T cells (x-axis), normalized to migration to positive control (chemokine mix of CCL2 (50 ng/ml), CCL4 (50 ng/ml), CCL19 (1000 ng/ml), CCL20 (100 ng/ml), CXCL10 (500 ng/ml), CXCL12 (100 ng/ml), CXCL13 (1000 ng/ml) and GM-CSF (500 ng/ml)) towards HPV-negative and -positive head and neck squamous cell carcinoma (HNSCC) cell lines (y-axis). Symbols represent experimental replicates from various human peripheral blood mononuclear cell (PBMC) donors. Dotted line indicates the mean plus two times the standard deviation (SD) of migration towards negative control (medium only). Data are presented as mean and error bars indicate standard deviations. When the average exceeds the dotted line, it is considered as migration. *P*-values were obtained by an unpaired non-parametric Kruskal-Wallis test with *** *p*<.001


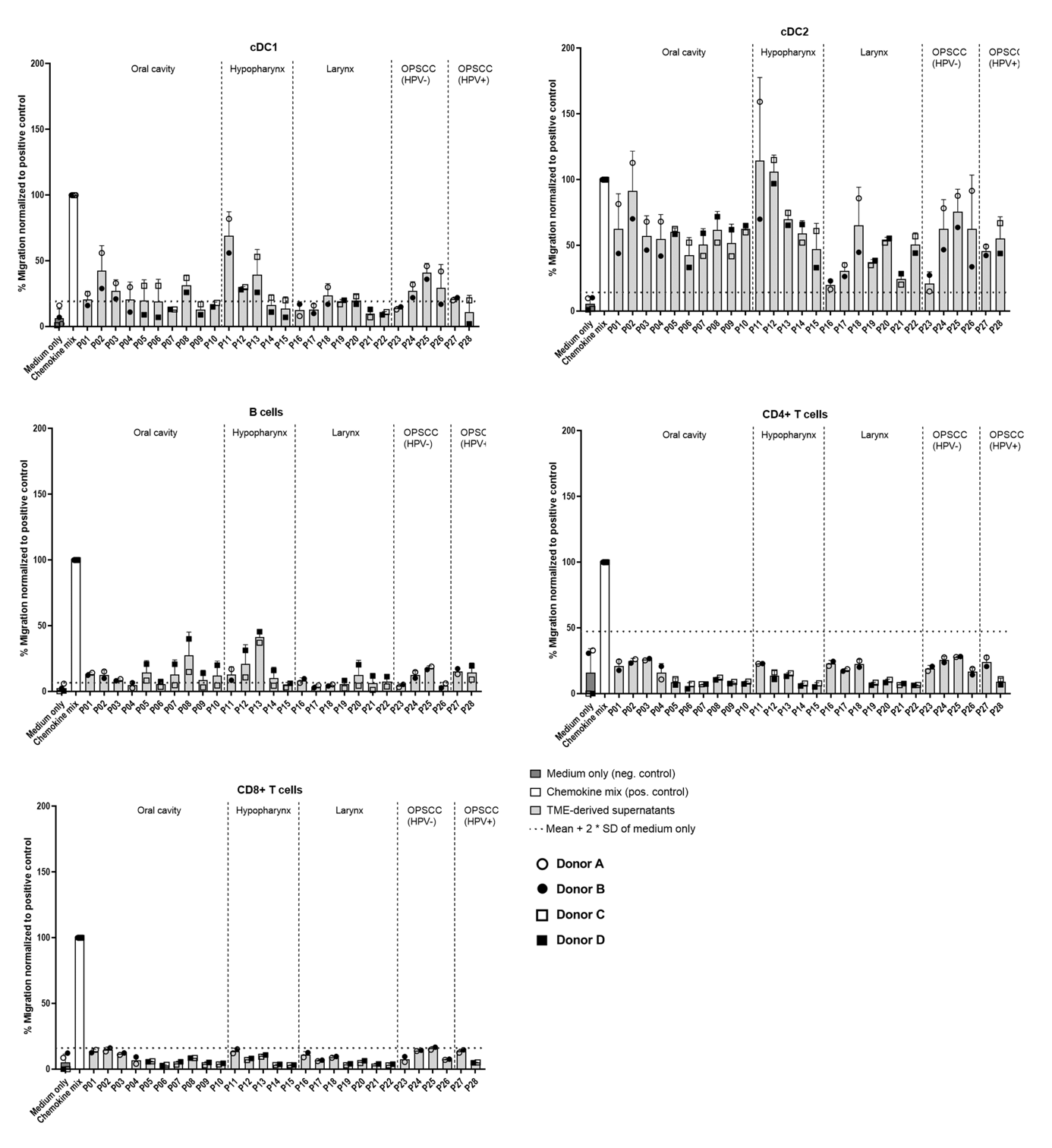


**Supplementary Fig 3.** **Migration of immune subsets towards tumor microenvironment (TME)-conditioned media.** Migration of conventional dendritic cells type 1 (cDC1), conventional dendritic cell type 2 (cDC2), B cells, CD4+ T cells and CD8+ T cells (y-axis), normalized to migration to positive control (chemokine mix of CCL2 (50 ng/ml), CCL4 (50 ng/ml), CCL19 (1000 ng/ml), CCL20 (100 ng/ml), CXCL10 (500 ng/ml), CXCL12 (100 ng/ml), CXCL13 (1000 ng/ml) and GM-CSF (500 ng/ml)) towards 24h conditioned medium derived from digested head and neck cancer specimen. Data presented per patient (x-axis), and are ordered based on HNSCC anatomical site (vertical dotted lines): oral cavity squamous cell carcinoma (SCC), hypopharynx SCC, larynx SCC, human papillomavirus (HPV)-negative oropharynx SCC (OPSCC) and HPV-positive OPSCC. Symbols represent experimental replicates from various human peripheral blood mononuclear cell (PBMC) donors. Horizontal dotted line indicates the mean plus two times the standard deviation (SD) of migration towards negative control (medium only). Data are presented as mean and error bars indicate standard deviations. When the average exceeds the dotted line, it is considered as migration.


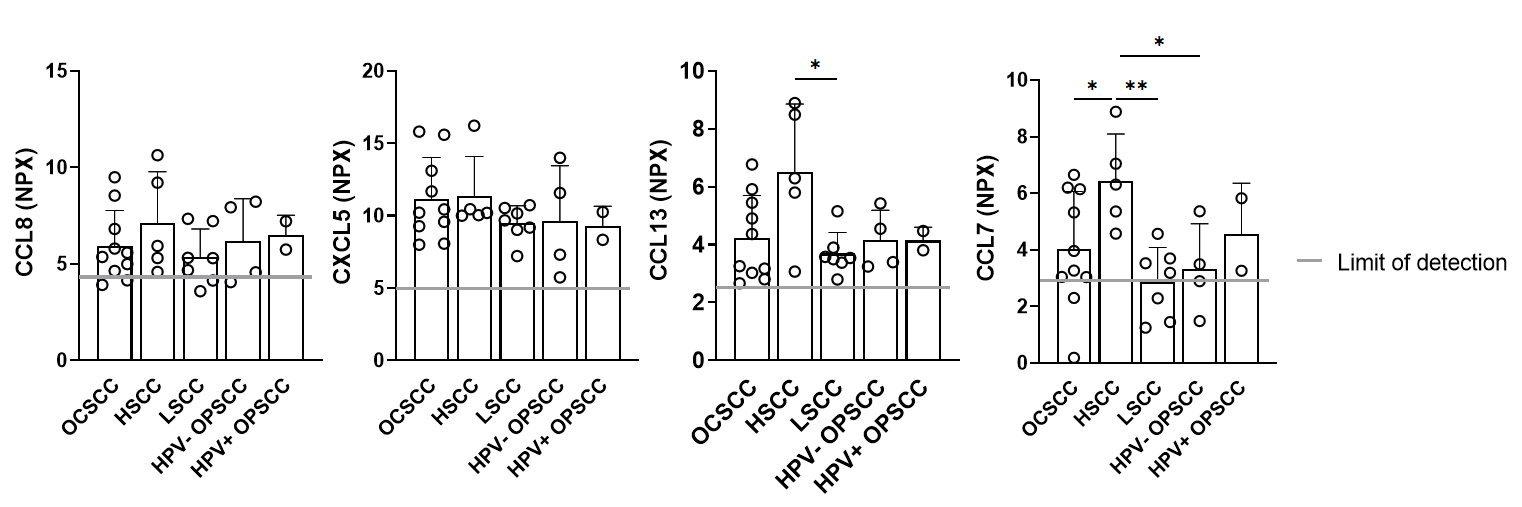


**Supplementary Fig 4. Protein levels in tumor microenvironment (TME)-derived secretomes by anatomical site and HPV involvement.** Levels of proteins measured by Olink Target Immuno-Oncology assay in normalized protein expression (NPX, y-axis) for TME-derived overnight secretomes from tumor specimen originating from oral cavity squamous cell carcinoma (OCSCC), hypopharynx SCC (HSCC), larynx SCC (LSCC) and human papillomavirus (HPV)-negative and -positive oropharyngeal SCC (OPSCC, x-axis). *P*-values were obtained by an unpaired non-parametric Kruskal-Wallis test with * *p*<.05, ** *p*<.01.


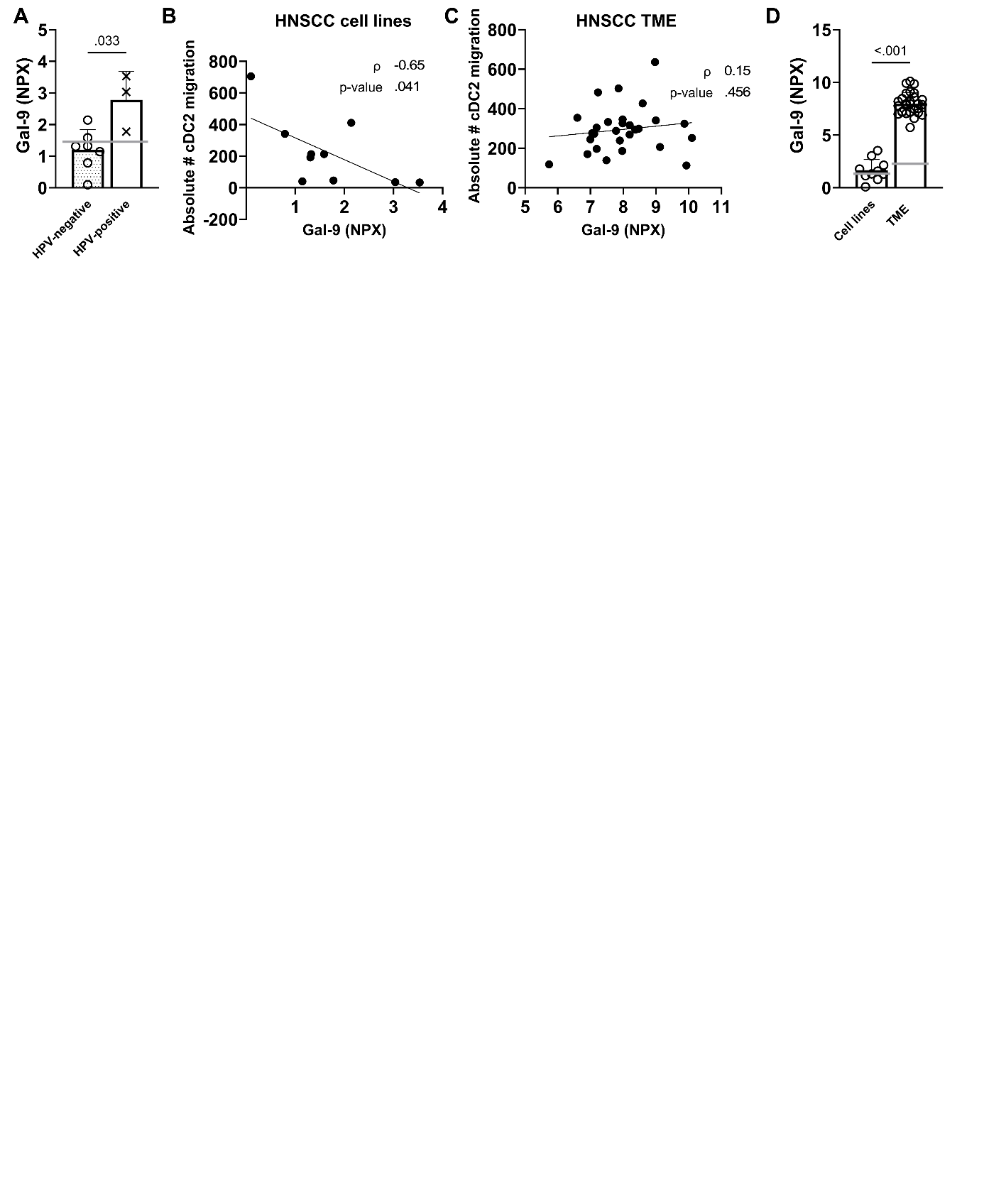


**Supplementary Fig 5. Galectin-9 (Gal-9) protein levels. (A)** Gal-9 levels in normalized protein expression (NPX, y-axis) between HPV-negative and HPV-positive cell lines. Limit of detection (LOD) with grey line indicated. *P*-value obtained by Mann-Whitney test and included in the figure. **(B-C)** Pearson correlation between Gal-9 protein levels in NPX value (x-axis) and cDC2 migration in absolute counts (#, y-axis) towards **(B)** secretome derived from head and neck squamous cell carcinoma (HNSCC) cell lines **(C)** and HNSCC tumor microenvironment (TME)-derived secretomes. *P*-value and correlation coefficient depicted in the graphs. **(D)** Comparison of Gal-9 protein level in NPX value (y-axis) between secretome derived from cell lines (left) and tumor microenvironment (TME) (right). Limit of detection (LOD) with grey line indicated. *P*-values obtained by Mann-Whitney test, included in the figure.


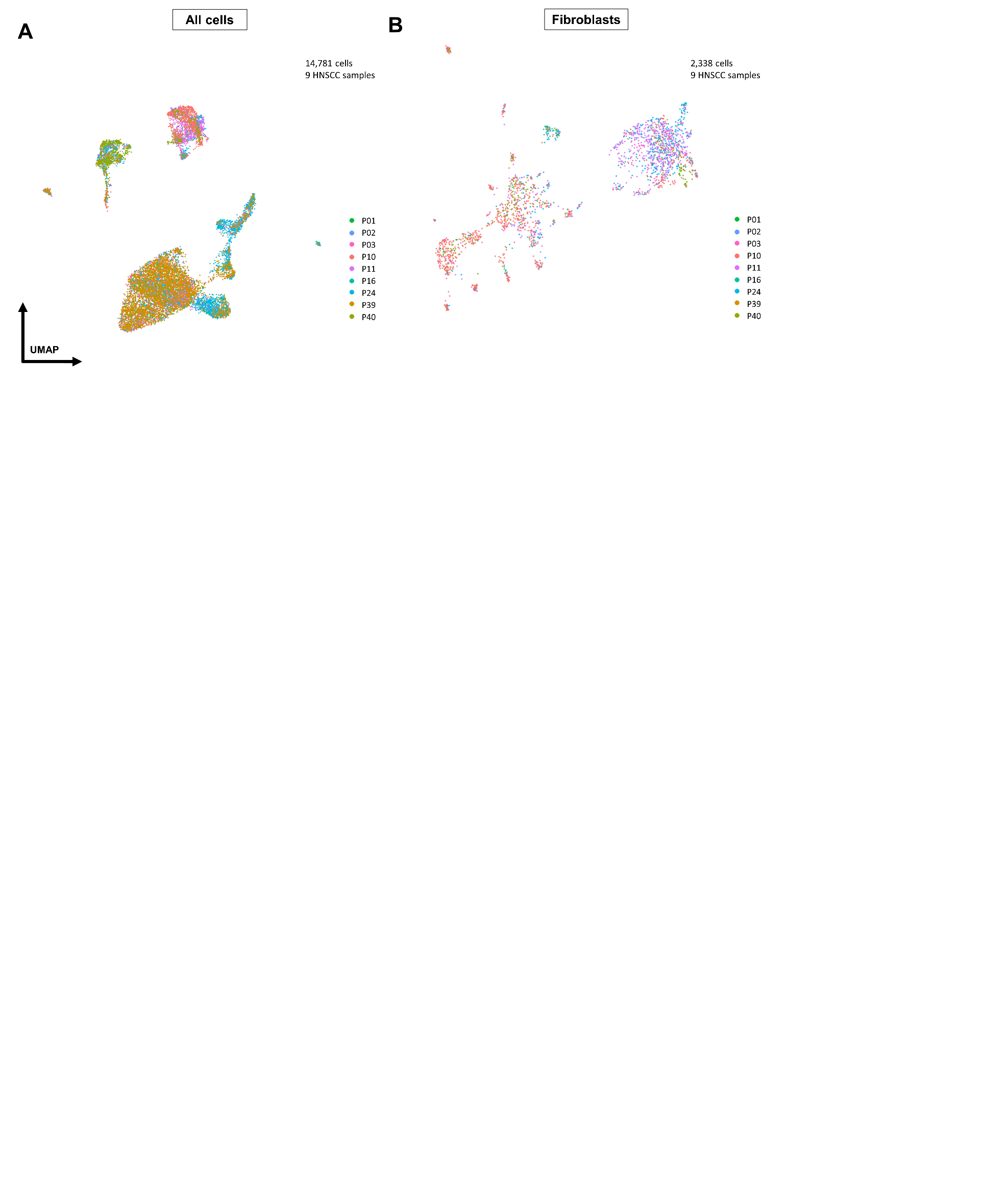


**Supplementary Fig 6.** Uniform Manifold Approximation and Projection (UMAP) grouped by patient (indicated with color) for **(A)** all cells and **(B)** fibroblasts.


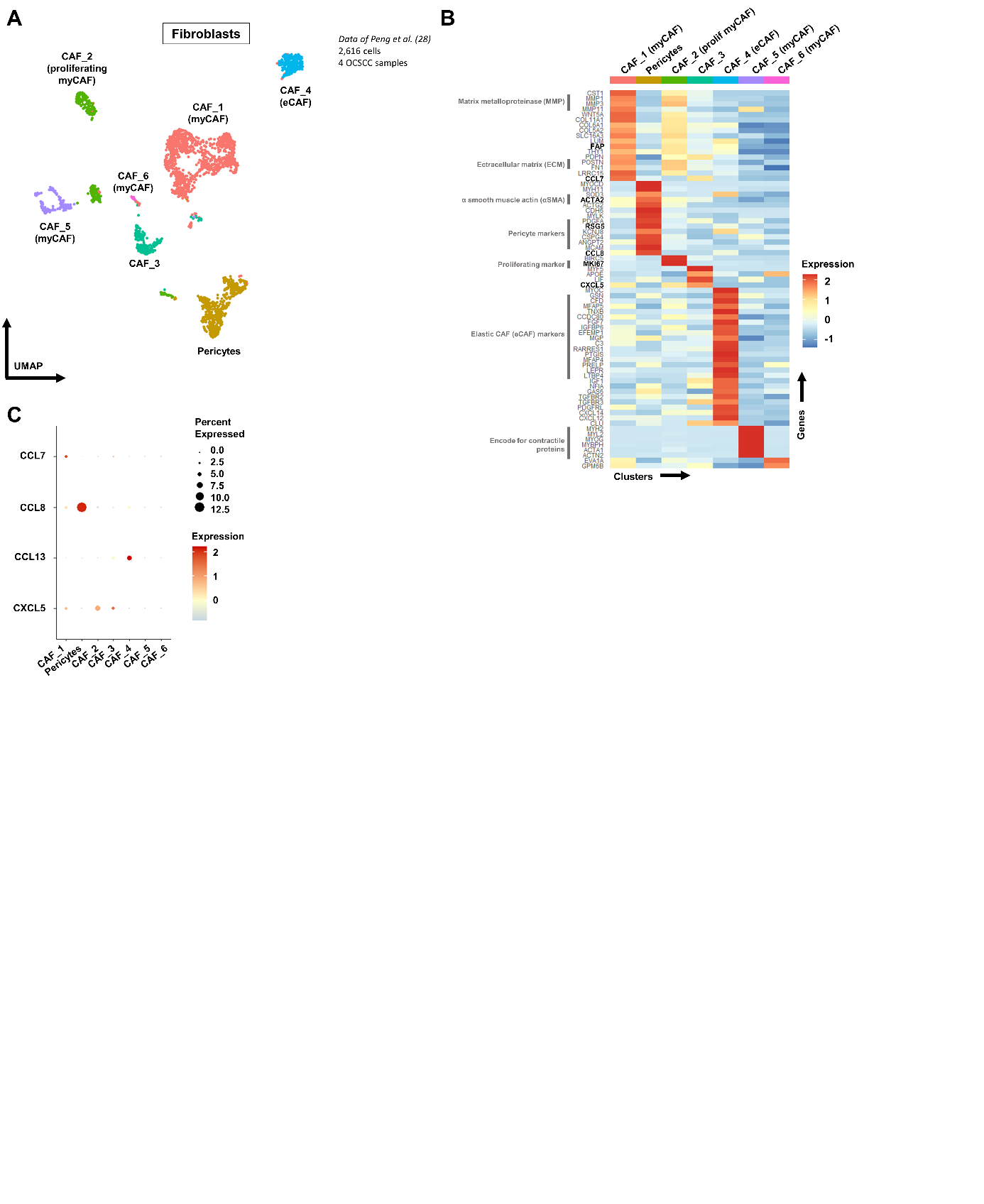


**Supplementary Fig. 7. Single cell RNA-sequencing (scRNA-seq) data of Peng *et al.* (31). (A)** Uniform Manifold Approximation and Projection (UMAP) of 2,616 cells from four oral cavity squamous cell carcinoma (OCSCC) specimen. **(B)** Average expression level of marker genes (y-axis) per subcluster (x-axis) **(C)** Average expression of CCL7, -8, -13 and CXCL5 (y-axis) in each subcluster (x-axis). Size of the circles represents percentage of cells with positive expression of the involved chemokines.


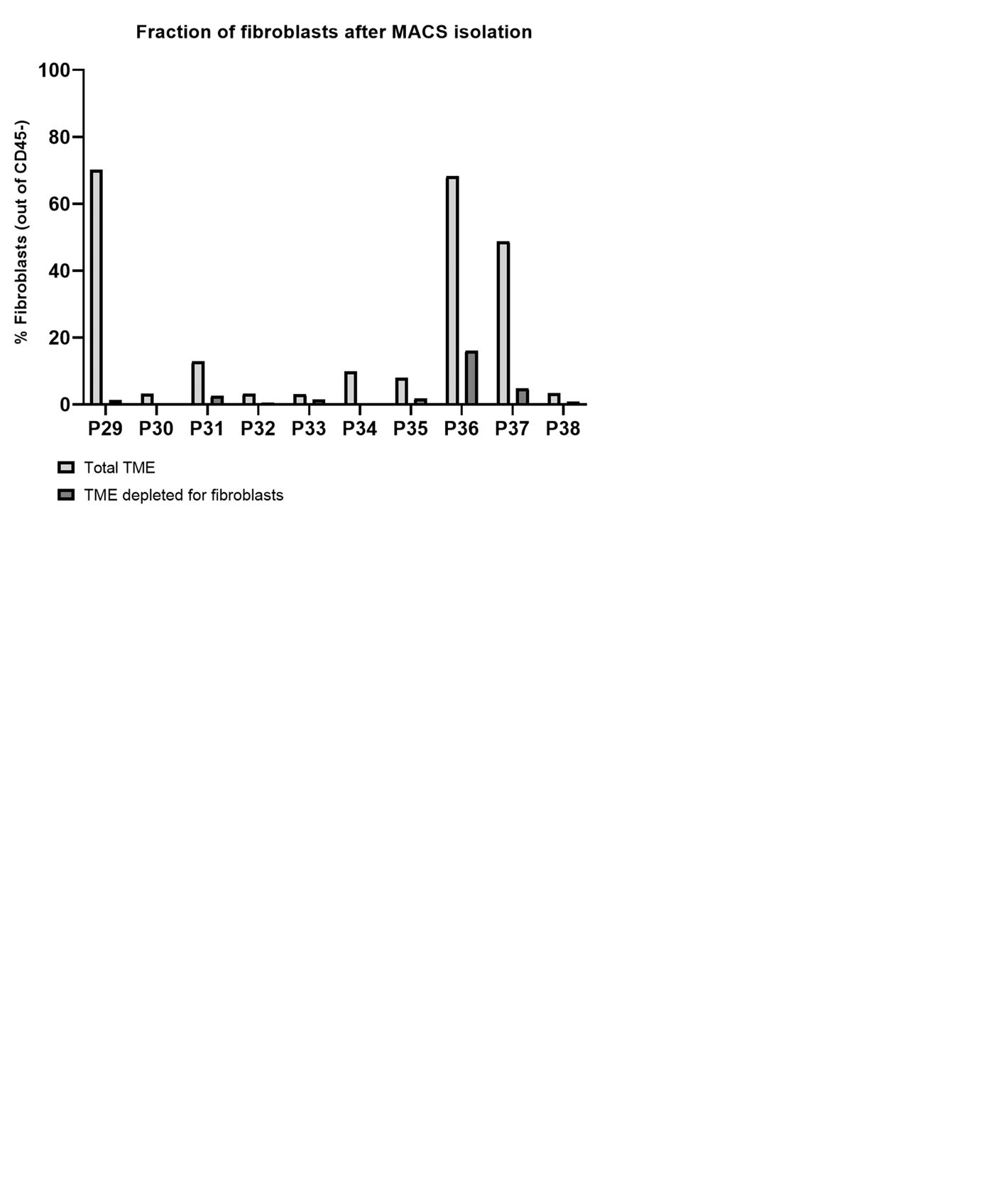


**Supplementary Fig 8.** Fraction of fibroblasts after magnetic-activated cell sorting (MACS) isolation (y-axis) per patients (x-axis). Fraction of fibroblasts in the total tumor microenvironment (TME) without MACS isolation in light grey and TME depleted for fibroblasts in dark grey.
